# An Unusual Cause of Acute Upper Gastrointestinal Bleeding: Acute Esophageal Necrosis

**DOI:** 10.1155/2016/6584363

**Published:** 2016-08-23

**Authors:** Nikhil R. Kalva, Madhusudhan R. Tokala, Sonu Dhillon, Watcoun-Nchinda Pisoh, Saqib Walayat, Vishwas Vanar, Srinivas R. Puli

**Affiliations:** ^1^Division of Gastroenterology and Hepatology, University of Illinois College of Medicine at Peoria, IL, USA; ^2^Department of Pharmacology and Pathophysiology, St. George's University School of Medicine, St. George's, West Indies, Grenada

## Abstract

Acute esophageal necrosis (AEN), also called “*black esophagus*,” is a condition characterized by circumferential necrosis of the esophagus with universal distal involvement and variable proximal extension with clear demarcation at the gastroesophageal junction. It is an unusual cause of upper gastrointestinal bleeding and is recognized with distinct and striking mucosal findings on endoscopy. The patients are usually older and are critically ill with shared comorbidities, which include atherosclerotic cardiovascular disease, diabetes mellitus, hypertension, chronic renal insufficiency, and malnutrition. Alcoholism and substance abuse could be seen in younger patients. Patients usually have systemic hypotension along with upper abdominal pain in the background of clinical presentation of hematemesis and melena. The endoscopic findings confirm the diagnosis and biopsy is not always necessary unless clinically indicated in atypical presentations. Herein we present two cases with distinct clinical presentation and discuss the endoscopic findings along with a review of the published literature on the management of AEN.

## 1. Case 1

A 45-year-old female presented to our institute after being found unresponsive along with clinical presentation of acute upper gastrointestinal bleeding with hematemesis and melena. Her medical history included active alcoholism, chronic acid reflux, and hepatitis C with chronic liver disease. She was seen previously for evaluation of dyspepsia 9 months ago with an unremarkable upper endoscopy with biopsies negative for celiac disease,* H. pylori*, and esophagitis. She was started on antisecr celiac trunk etory therapy.

**Figure 1:**
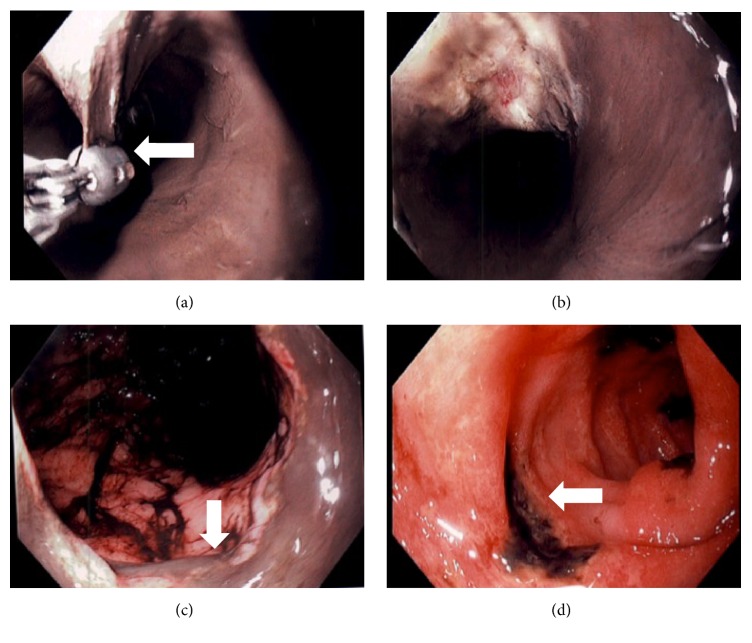
Images demonstrating diffuse mucosal necrosis extending from the mid to distal esophagus in circumferential fashion. A biopsy forceps showing mucosal dissection (UL marked with arrow) with exposure the muscle layer (UR). Stomach demonstrating old digested blood throughout (LL) and discrete duodenal ulcers (LR marked with arrow) with ulcer base coated with blackish material suggesting ischemic changes.

She was obtunded, with icteric sclera, malnourished with BMI of 17, and hypothermic with rectal temperature 34.4°C (94°F) and sustained systemic hypotension with blood pressure of 57/46 mmHg. She underwent rapid sequence intubation for airway protection. Neurologic exam was nonfocal devoid of meningeal signs. Cardiac exam was unremarkable with normal heart sounds. Coarse breath sounds bilaterally with wheezing. Abdomen exam was soft with sluggish bowel sounds. Labs are in Table 1. She was admitted to medical ICU and started on high dose PPI and empiric broad spectrum antibiotics were given. Emergent EGD was performed which revealed circumferential black esophageal mucosa extending from the middle and distal portions with sharp demarcation at the gastroesophageal junction (Figure 1). The mucosal layer appeared separate from the underlying muscular layer suggesting intramural esophageal dissection. A biopsy forceps was used to grasp the mucosa to confirm the findings (Figure 1). The stomach appeared normal with old blood that was washed. The duodenal examination revealed large ulcerations extending from the bulb and into the examined portion of the third segment of the duodenum. She remained critically ill and was supported with inotropic agents, bowel rest, PPI, and empiric broad-spectrum antibiotics. Despite aggressive medical management, which included continuous venovenous hemofiltration, ventilator support, and goal directed resuscitation, the patient died on day #3 from multiorgan failure which included acute renal failure, acute respiratory distress syndrome, and disseminated intravascular coagulation likely from sepsis.

## 2. Case 2

**Figure 2:**
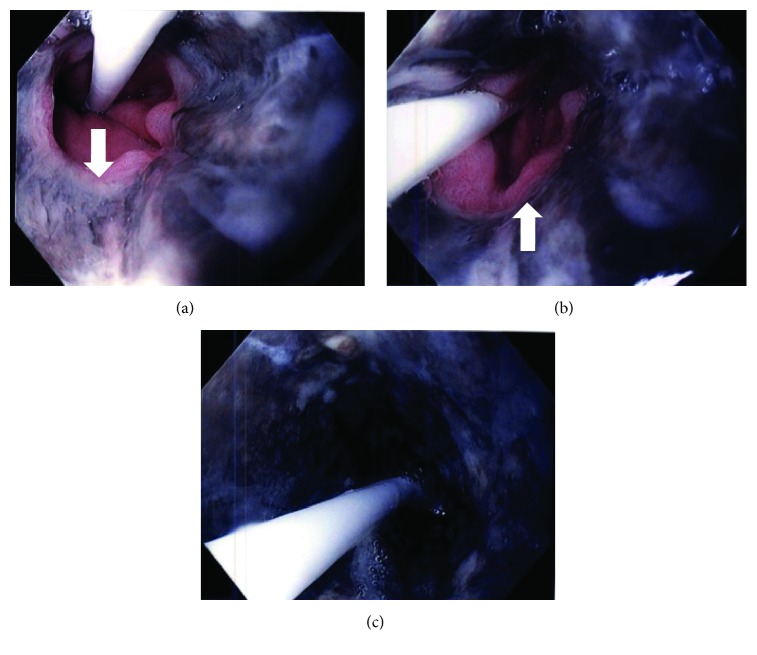
Images demonstrate circumferential esophageal necrosis extending from the mid esophagus (LL) to the distal esophagus with clear demarcation at the gastroesophageal junction (UL and UR with arrow). A transesophageal pressure catheter is seen in the images with distal end in the stomach.

A 66-year-old female was admitted for evaluation of nausea, coffee ground emesis, and retrosternal and epigastric pain. Her medical history is significant for type II DM, coronary artery disease with CABG, essential hypertension, and hyperlipidemia. On exam she was frail and ill appearing with a temperature of 32.6, BP 170/90, pulse 112, and respiratory rate 28. She appeared confused with slurred and delayed speech. There were no focal deficits and the rest of the exam was unremarkable. An NG was placed with return of large amount of coffee ground aspirates. Lab revealed marked hyperglycemia with glucose 696 g/dL, bicarbonate 5, pH 6.8, CO_2_ 5, BUN 41, Cr 1.74 mg/dL, procalcitonin 1.83, WBC 20.26, and hemoglobin 8.5 g/dL. She was started on insulin infusion and fluids for diabetic ketoacidosis and high dose PPI for upper gastrointestinal bleed. An urgent upper endoscopy revealed scattered ulcerations of the proximal esophagus with circumferential blackish extending from the mid to distal esophagus consistent with AEN (Figure 2). There was a clear demarcation at the GEJ. The rest of the endoscopic examination was normal. There was no evidence of active bleeding. The patient was discharged on day 5 without any additional complication.

## 3. Discussion

Acute esophageal necrosis (AEN) or black esophagus is identified on endoscopy with presence of diffuse and circumferential blackish discoloration, with a sharp demarcation at the Z-line of the gastroesophageal junction. The distal esophagus is involved in almost all the cases with variable extension proximally. Goldenberg et al. first reported it in the 1990s and the term acute necrotizing esophagitis was used to describe the endoscopic findings [1]. Though the exact incidence remains unknown, various authors have suggested an incidence of <0.5% amongst endoscopy series suggesting rare clinical presentation [2]. AEN is commonly identified in older age group with a mean age of 67 years. It usually presents as acute upper gastrointestinal bleeding with clinical manifestation of hematemesis or melena and upper abdominal pain [3]. Biopsy is not indicated in typical cases unless atypical infection such as CMV or herpes is suspected usually in the setting of profound immunosuppression. Primary infections are implicated only in a small fraction of the patients, usually in immunocompromised state [3]. Histopathology if performed demonstrates extensive mucosal necrosis with ulceration and hemosiderin deposits. Necrosis may extend into deeper layers involving the muscularis propria with vascular thrombosis suggesting ischemic injury. Special staining with Perl's Prussian blue and Fontana-Masson is negative and helps exclude iron pill injury and esophageal melanocytosis, respectively [4].

The pathophysiologic mechanism responsible for AEN remains poorly understood, but the insight from the careful study of published case reports suggests a two- or even multihit process. While ischemia plays an important risk factor other recognized associations include gastric outlet obstruction, chronic reflux, diabetic ketoacidosis, severe malnutrition, alcoholism, and congestive heart failure. The preferential and universal involvement of the distal esophagus could suggest “water shed” pattern of ischemia resulting from a low flow state with decreased perfusion pressure. Occlusion of the celiac trunk and its branches or dissection of the thoracic aorta could compromise the vascular supply with similar presentation. As in our patients above, the clinical presentation with sustained hypotension could explain this theory. Alternative mechanisms of injury in patients that lack clinical presentation preceded by hypotension include massive exposure to gastric acid reflux with prolonged exposure of the esophageal mucosa [5, 6]. This could be seen in patients that present with gastric outlet obstruction or volvulus.

The prognosis is variable with as much as 32% dying after the diagnosis is established, though the cause is likely due to overall global illness state rather than AEN [7]. This finding raises the question of AEN as a systemic manifestation than an isolated condition. There are no established guidelines on the management due to paucity of high quality studies. Supportive care with expectant management is considered acceptable with restoration of systemic circulation and organ perfusion, antisecretory therapy, and antibiotics. It remains unclear if there is a role for antibiotics, but initiation is considered acceptable especially in patients presenting with clinical signs of sepsis. Antivirals or antifungals would be indicated in patients with identified viral or fungal elements, especially in special risk groups such as immunocompromised hosts. Bowel rest with high dose proton pump inhibitors is usually initiated at the time of diagnosis and is continued for 48–72 hours to further decrease esophageal acid exposure. Surgery would be indicated if there was evidence of perforation with full-thickness necrosis or mediastinitis recognized on imaging studies or presence of subcutaneous emphysema. A repeat endoscopy is also warranted to look for strictures, which can be seen in 25% of the patients that could be treated with endoscopic therapy [8].

In conclusion, AEN is a rare cause of acute upper gastrointestinal bleeding with typical endoscopic findings with increased risk of in-hospital mortality. While the disease specific mortality and need for esophageal surgery are low, the overall mortality remains high as suggested by comorbidities.

## Figures and Tables

**Table 1:** 

	Case 1	Case 2
Age; sex	45 yr; female	66 yr; female
Clinical presentation	Septic shock, acute upper gastrointestinal bleeding	Diabetic ketoacidosis, coffee ground emesis, and epigastric pain
Associated condition	Malnutrition, GERD, chronic liver disease, and alcoholism	Malnutrition, coronary artery disease, hypertension, and diastolic heart failure
Hemoglobin (g/dL)	9.5	8.5
Creatinine (mg/dL)	7.1	1.9
Lactic acid (<2.1 mmol/L)	10	1
Procalcitonin (>0.5 ng/mL is positive)	19.7	1.8
Endoscopic features	Circumferential black esophagus from mid to distal third w/intramural esophageal dissection Duodenal ulcerations with low risk stigmata for bleeding	Isolated circumferential black esophagus from mid to distal third
Etiology	Malnutrition, alcoholism, chronic acid reflux, sepsis, and ischemia	DKA, systemic hypotension, ischemia, and possibly low flow ischemia
Clinical outcome	Deceased	Alive at discharge without short-term complications

## References

[1] Goldenberg S. P., Wain S. L., Marignani P. (1990). Acute necrotizing esophagitis. *Gastroenterology*.

[2] Augusto F., Fernandes V., Cremers M. I. (2004). Acute necrotizing esophagitis: a large retrospective case series. *Endoscopy*.

[3] Gurvits G. E., Shapsis A., Lau N., Gualtieri N., Robilotti J. G. (2007). Acute esophageal necrosis: a rare syndrome. *Journal of Gastroenterology*.

[4] Chang F., Deere H. (2006). Esophageal melanocytosis: morphologic features and review of the literature. *Archives of Pathology and Laboratory Medicine*.

[5] Burtally A., Gregoire P. (2007). Acute esophageal necrosis and low-flow state. *Canadian Journal of Gastroenterology*.

[6] Grudell A. B. M., Mueller P. S., Viggiano T. R. (2006). Black esophagus: report of six cases and review of the literature, 1963–2003. *Diseases of the Esophagus*.

[7] Moreto M., Ojembarrena E., Zaballa M., Tanago J. G., Ibanez S. (1993). Idiopathic acute esophageal necrosis: not necessarily a terminal event. *Endoscopy*.

[8] Gurvits G. E., Cherian K., Shami M. N. (2015). Black esophagus: new insights and multicenter international experience in 2014. *Digestive Diseases and Sciences*.

